# Support for a right-wing populist party and subjective well-being: Experimental and survey evidence from Germany

**DOI:** 10.1371/journal.pone.0303133

**Published:** 2024-06-26

**Authors:** Maja Adena, Steffen Huck

**Affiliations:** 1 Research Unit Economics of Change, WZB Berlin, Berlin, Germany; 2 Department of Economics, UCL London, London, United Kingdom; Georg-August-Universität Göttingen: Georg-August-Universitat Gottingen, GERMANY

## Abstract

With the rise of populism in many countries, including Germany, it is more important than ever to better understand the causes and consequences of populist support. Using two experiments within the context of a large panel survey, we study how support for the German right-wing populist party *Alternative für Deutschland* (AfD) is associated with subjective perceptions of personal and financial well-being. In both experiments, we rely on priming the identity of AfD supporters, once in a controlled manner and once in a natural setting. We document a causal relationship from AfD support to diminished well-being for new and marginal AfD supporters. Our findings challenge the prevailing assumption that causality moves unidirectionally, from life dissatisfaction to support for populist parties, and suggest that early interventions focusing on positive messages are particularly promising to win voters back into the mainstream.

## Introduction

With the surge of populism in various countries, including Germany, it is now more crucial than ever to gain a better understanding of the causes and consequences of populist support. At the beginning of 2024, the right-wing populist party *Alternative für Deutschland*, AfD, surpassed 20% in national German polls [1, 2]. Remarkably, in all East German states except Berlin, it became the strongest party [3].

What do voters experience when they turn to a party such as the AfD? This is an important question as any change in the emotional states of voters who turn to an extremist populist party might have repercussions for how to optimally defend liberal democracy. Is it a surge of happiness that voters experience by finally taking a stance against the mainstream? There is a large body of literature suggesting that this might be the case. Taking a stance goes along with establishing a sense of self-determination and agency, which are seen as important for well-being (see, for example, [4] or [5], who build on self-determination theory). It may also reduce cognitive dissonance [6] and induce a sense of achievement through embracing a goal, which has also been shown to boost happiness (see, for example, [7] or [8]). Finally, taking a stance can serve as a successful strategy for coping with stress and increase well-being through stress alleviation [9, 10].

**Fig 1:**
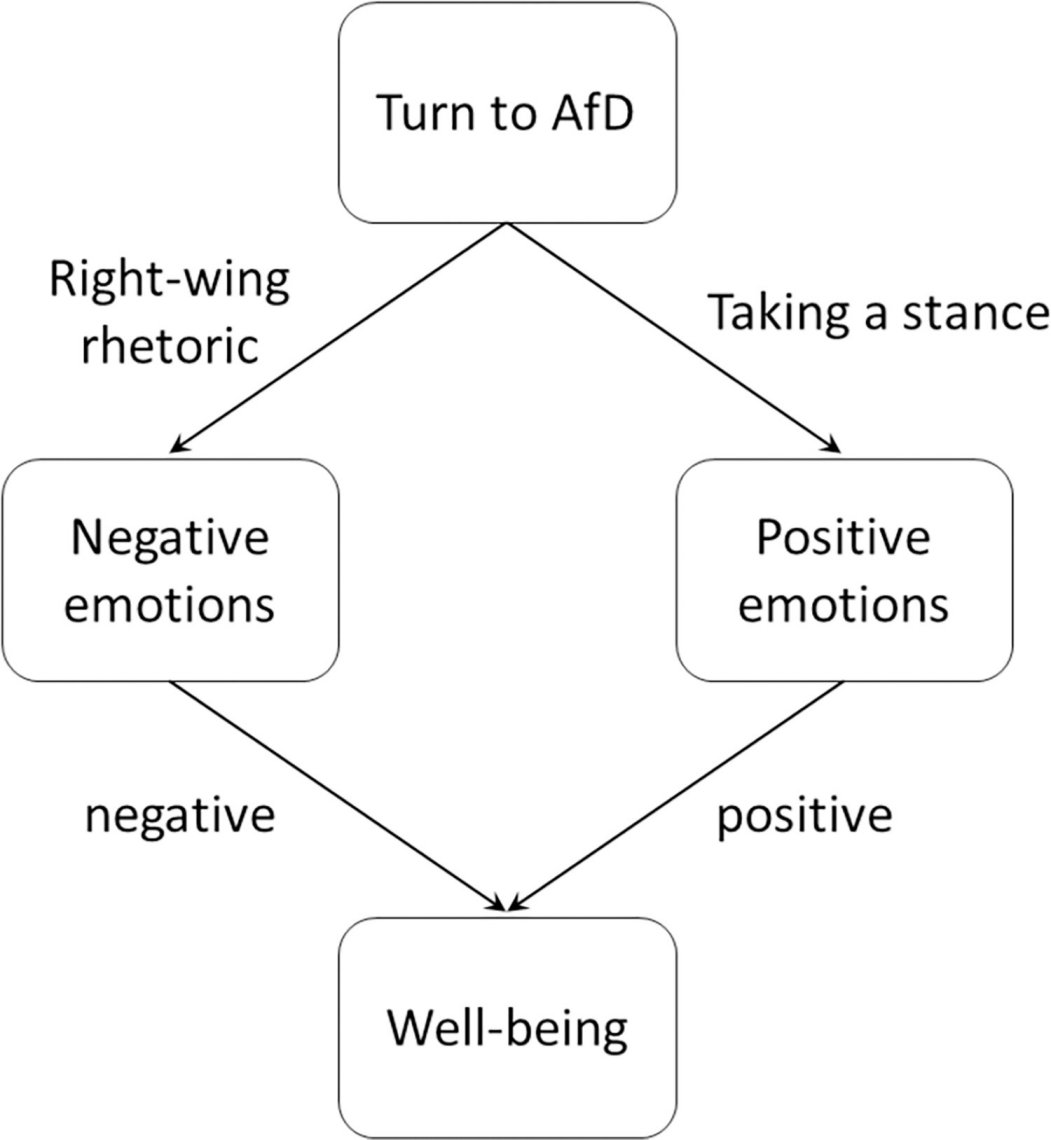
Two potential paths by which the decision to support the AfD can affect individual well-being.

But there are also reasons to believe that voters might experience deteriorations in well-being when they swear allegiance to a populist party such as the AfD. Right-wing movements thrive on a rhetoric of negativity, swamping their supporters with negatively framed topics and news [11–14]. Also, right-wing populist communication strategies strongly rely on negative emotions [15] and fear [16], and populist messages are often characterized by assigning blame to elites in an emotionalized way [17]. Such negative messages are likely to “infect” recipients through emotional contagion, a phenomenon well established in the literature [18, 19] that has negative repercussions for well-being. For example, exposure to especially negative news on TV has been shown to have an adverse effect on viewers’ mental well-being, triggering anxiety and sadness [20, 21], and the reporting of mass violence has been shown to induce significant distress [22]. Even with short exposure to news on the 9/11 attacks, individuals reported negative emotions, such as shock, fear, and anger [23–25], while as an effect of long exposure, people became more anxious and emotional [26–28] and reported higher levels of posttraumatic stress disorder symptoms [29]. In a similar vein, recent studies relating to COVID-19 have shown that exposure to negative news about the pandemic triggered depression symptoms [30], increased self-reported mental distress [31], and was correlated with more severe self-reported mental health symptoms [32] and reductions in positive affect and optimism [33]. On the other hand, news stories featuring others’ kindness have been shown to have the potential to undo the aversive effects of negative news [34]. In the party context, which we study here, negative populist appeals have been shown to trigger negative emotions in their supporters [35]. We summarize the two potential paths by which the decision to support the AfD can affect individual well-being in Fig 1.

Having good reasons for expecting effects in either direction, we embark on providing causal evidence for the consequences of support for a right-wing party on well-being. Understanding the psychological consequences of such a choice is important for at least two reasons: They may inform the content and timing of interventions designed to win voters back into the mainstream. If voters experience surges of happiness when joining the populists’ ranks, promising interventions will ultimately have to tackle the topics championed by the populists and will have to wait until initial surges in well-being ebb. In contrast, if voters experience a deterioration in well-being, effective interventions will embrace different topics not laden with the same negativity and will be swift.

We examine the consequences of supporting a right-wing populist party on well-being, utilizing two experiments in the context of a panel survey in Germany. Both experiments rely on identity priming, but they differ in the way the priming arises. In one experiment, the priming is fully controlled through a module within the survey; in the other, we exploit naturally occurring differences in exposure to news about the AfD over time. Both experiments exhibit the same robust pattern: Voters with a new and not-yet-fully-formed identity as AfD supporters who are reminded of their allegiance to the AfD report diminished well-being. The effect is weaker (in the controlled experiment) or reversed (in the natural experiment) for other AfD supporters. This suggests that it is the initial *decision* to support a right-wing party that has the most profound negative effect on well-being.

## Materials and methods

### Data

We collected data from four waves of a survey gathered for us by YouGov Germany in 2019, 2020, and twice in 2021—before and after the federal elections. In wave 1, in December 2019, we sampled 5,078 respondents. These were composed of a representative sample of German voters consisting of 4,094 respondents, to which we added 984 additional randomly drawn AfD supporters based on self-reported voting in the 2017 federal elections recorded by YouGov. This generated a total of 1,397 voters who voted for the AfD in 2017. We oversampled AfD voters in order to increase the power for tests that compare AfD supporters with nonsupporters and to increase the number of those respondents who switched party allegiance. In wave 2, in November/December 2020 we re-contacted all respondents from 2019 and received responses from 3,864 participants, or 76% of the full 2019 sample. In this wave, we exploit the fact that the AfD federal party convention took place on November 28–29, 2020, right in the middle of our sampling window (November 23–December 14, 2020). This furnishes us with a natural experiment, on which more details will follow below. In wave 3, from the end of August until a few days before the federal elections held on September 26, 2021, we re-invited all previous participants, of whom 3,359 took part again, and we added a new fresh sample, consisting of 1,451 2017 AfD voters and 251 non-AfD voters, generating a total of 5,061 participants. In wave 4, in November/December 2021, we invited all participants from the three previous waves and achieved a response rate of 86%, generating a total of 4,342 participants. This wave contained an experimental module, which is explained below in more detail.

A WZB Research Ethics Review (2021/0/130) has been obtained for the experimental part of the study. Data collection was done by YouGov. YouGov obtained informed consent in written form when the participants registered with the platform. Due to the collection of answers pertaining to questions about political orientation and health, YouGov obtained additional written consent in each wave of the survey, and the participants always had the option to withdraw from the survey or skip sensitive questions.

#### AfD support measurement

We collected information on retrospective voting behavior in the parliamentary elections in 2017 and 2021 and on voting intentions using the standard question: “If the federal elections were next Sunday, which party would you vote for?” This allows us to distinguish between new and previous AfD voters. As answers related to AfD support might be affected by experimental conditions, we define our target groups for the controlled experiment (new versus other AfD supporters) on the basis of responses in the previous wave. In the controlled experiment, we exploit a question from the previous wave that asked about participants’ voting intentions in the upcoming parliamentary elections and asked when participants made their decision. We classify those who indicated intentions to vote for the AfD and stated that they did not make this choice a long time ago as new AfD voters. In the natural experiment, we do not have a similar question from the previous wave but exploit the previous wave’s questions about the extent of support for the AfD party manifesto, classifying those with limited support for the manifesto as marginal AfD voters, who we then compare to other AfD voters.

#### Well-being measurement

The literature on populism has tended to focus on measures of income or subjective measures of personal dissatisfaction at an absolute level (through elicited agreement with statements such as “my life is close to ideal” or “I am satisfied with my life”) or through explicit comparisons with others. We instead measure well-being by focusing on *internal* change, asking respondents to compare their current situation with the previous year or to measure their expectations for the future compared with the present. We made this choice following previous research that has shown that reference points are useful in increasing informativeness [36, 37]. Without explicitly providing reference points, individuals might draw on reference points that are unknown to the researcher, making comparisons difficult and introducing noise. Furthermore, prior research has shown that people make intrapersonal comparisons more often than interpersonal ones and that they consider the former to be more important [38]. In addition, Germany is a notable exception with respect to the “losers of globalization” theory [39]: for the German AfD, there is broad agreement in the literature (which relies on interpersonal comparisons) that neither variables related to low socio-economic status, such as education, income, occupational prestige, or unemployment, nor subjective perceptions of one’s adverse personal (economic) situation are related to AfD support [40–45]. An exception is Pesthy et al. [46], who, however, interestingly ask respondents about their “current” economic situation, thus also encouraging an intrapersonal comparison. We conjectured that measurements relying on interpersonal comparisons may not capture feelings of misery that cannot be justified by objective comparisons with others and that this may plausibly change when we ask about purely internal comparisons.

More specifically, in waves 2 to 4, we asked participants about their personal well-being and about their financial well-being compared to the previous year as well as about the outlook for the coming year (“When you think about the past year, how are you today personally [financially]? Better than in the past year; as well as in the past year; worse than in the past year” and “When you think about the coming year, how do you estimate you will be doing personally [financially] in comparison to today? Better than today; the same as today; worse than today.”). Based on their responses, we created dummy variables indicating whether participants judge their situation to be worse than in the year before or expect it to get worse in the next year. The average of those four variables is our main indicator of well-being.

### Other variables

We also collected data on general political attitudes, place of residency, socio-economic status, including household size and income, and financial well-being, and we use them as control variables. Table A1 in the S1 File provides some summary statistics by wave.

## Analysis

### Identity priming and subjective well-being—the controlled survey experiment

We implemented a survey experiment priming political identity in wave 4 of our survey. To assign the participants to a particular party priming module, we asked which party they voted for in the parliamentary elections of 2021, which party they could imagine supporting, and which party program they partly or fully support. Those who had voted for the AfD, who could imagine voting for the AfD, or who support the AfD party program fully or partly were primed with their AfD identity. Hence, we do not just define AfD supporters as past voters but also as individuals who support the party and could imagine voting for the AfD. The remaining individuals were primed with questions on the party they indicated having voted for in 2021.

In the priming module, participants were forced to think deeply about the party they now support. More specifically, participants were presented with seven key manifesto items and asked to which extent they agreed with those. They were also presented with names of three key party politicians and were asked how good they find their work and whether they wished they had more influence. In addition, they were asked to specify further reasons for their support of the respective party in an open text field. The exact formulation of those questions (translated into English) and basic summary statistics can be found in the Section B in S1 File.

Importantly, half of the respondents received this module before the well-being section, while the other half received these questions afterwards.

Among the respondents who received the questions pertaining to the AfD, there was close to 100% support for border controls and the deportation of refugees who have committed crimes as well as for the introduction of referendums in Germany according to the Swiss model. The other program points received support of 76–79%, with the exception of the proposed COVID-19 policy, which involved abandoning all measures completely; this attracted more limited support of 60%. The remaining respondents received the same type of questions but pertaining to the parties they voted for. The advantage of this approach is that we have full control over who is treated and who is not and who is primed with AfD topics versus another party’s topics.

For the purpose of the analysis, among the individuals primed with the AfD, we distinguish between new and other AfD party supporters. As the distinction between new and other AfD party supporters could potentially be influenced by the order in which the questions in wave 4 were asked, we rely on wave-3 responses to classify individuals. Among individuals primed with AfD topics, we define a new AfD supporter as somebody who stated in wave 3 that they were going to vote for the AfD in the upcoming parliamentary elections but that they had not made this decision long ago. There are 252 individuals that fulfill these criteria (and for whom baseline characteristics are available). The remaining individuals, who received the priming module, are classified as other AfD supporters. There are 1,471 individuals in this category. The remaining individuals—those who were not primed with AfD topics—are classified as nonsupporters. This group consists of 2,297 individuals.

We created a “priming” dummy equal to one for participants who were confronted with the priming block on politics before the block on well-being. We also created a “no priming” dummy for participants who were confronted with the block on wellbeing before the block on politics. As for the measure of well-being, we created a variable indicating average well-being (higher values correspond to lower well-being), which is calculated as the average of nonmissing values of all four main well-being questions.

We hypothesize that the well-being of AfD supporters will either be positively or negatively affected when they are confronted with AfD topics and that new supporters will be affected most. The first part of the hypothesis builds on the literature presented above. Taking a stance could affect well-being positively, or populist rhetoric centered around negative emotions could result in emotional contagion. The second part of the hypothesis builds on findings in Hameleers et al. [17], who find that emotionalized blame attributions in the context of populist communication show the strongest persuasiveness for citizens with weaker identities. It is also in line with Cohen [47], who find that participants with weaker party identification tend to be more influenced by party priming manipulations.

In order to better structure the upcoming analysis, we formulate the following testable null hypotheses:

H1: There is no priming effect for new AfD supporters.
H2: The priming effect for new AfD supporters is not different from the effect for other AfD supporters.
H3: The priming effect for new AfD supporters is not different from the effect for supporters of other parties.
H4: There is no overall priming effect.

**Fig 2:**
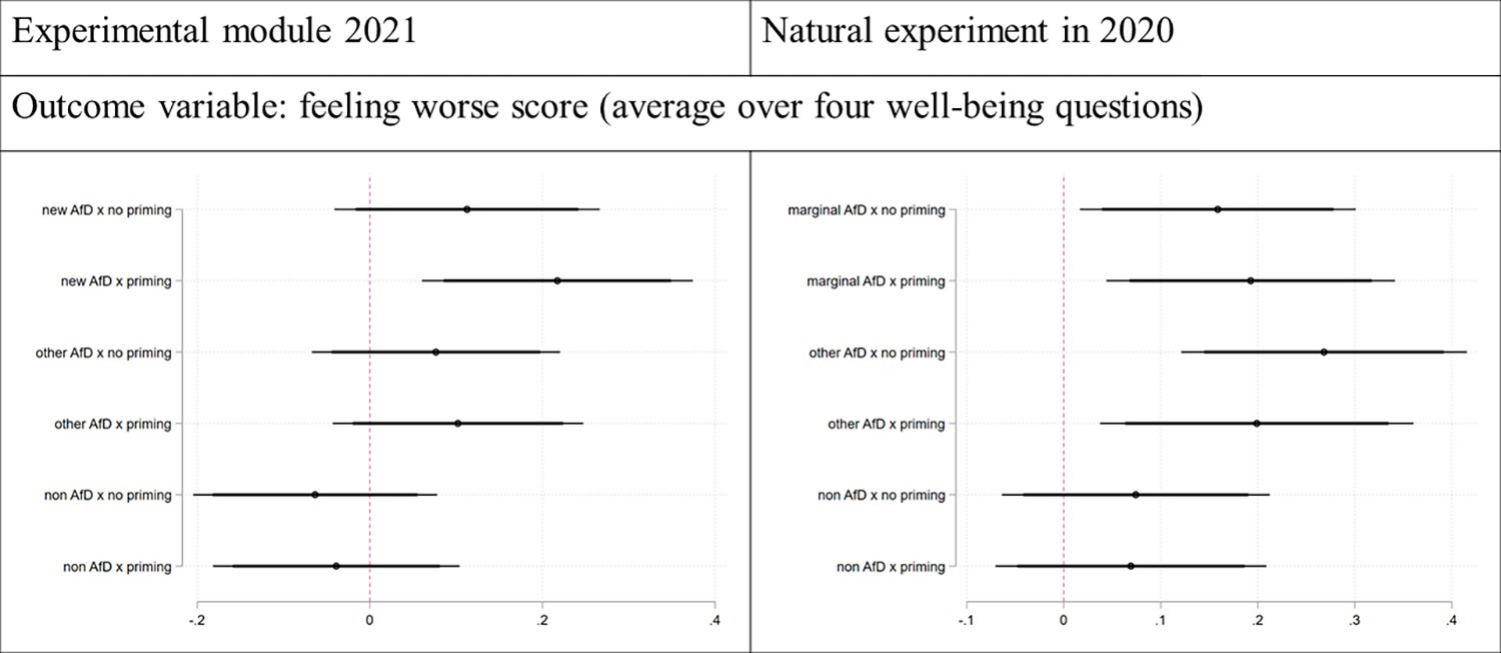
Causal effects of priming political identity in two experiments: Experimental module in Wave 4 and natural experiment in Wave 2. Coefficient estimates from Table 1; thick lines—90% confidence intervals, thin lines—95% confidence intervals.

We present our results in Table 1 (Column I) and visualize them in Fig 2 (left panel, Column II; the right panel of the Figure presents the results from the natural experiment, which will be explained in the next section). More specifically, we present coefficient estimates from an OLS regression without a constant, where each AfD status dummy (new, other, non-AfD) is interacted with the treatment dummies for priming or no priming. We also include a baseline set of control variables. This gives us the means for all status-treatment combinations that account for corrections for any differences in observables between those groups. At the bottom of Table 1, we provide test statistics for the above four hypotheses.

**Table 1:** Causal effects of priming political identity in two experiments: Experimental module in Wave 4 and natural experiment in Wave 2.

		Experimental module in 2021	Natural experiment in 2020
Outcome variable:		feeling worse score (average over four well-being questions)	
		I	II
new (marginal) AfD x no priming	A	0.113 (0.078)	0.159** (0.073)
new (marginal) AfD x priming	B	0.217*** (0.080)	0.193** (0.076)
other AfD x no priming	C	0.077 (0.073)	0.268*** (0.075)
other AfD x priming	D	0.102 (0.074)	0.199** (0.082)
non AfD x no priming	E	-0.063 (0.072)	0.074 (0.070)
non AfD x priming	F	-0.039 (0.073)	0.069 (0.071)
vaccination block		-0.013 (0.011)	
age		0.012*** (0.003)	0.008*** (0.003)
age * age		-0.000*** (0.000)	-0.000** (0.000)
eastern German states dummy		-0.012 (0.014)	-0.027* (0.016)
Berlin dummy		0.007 (0.027)	0.009 (0.031)
suburban dummy		0.008 (0.014)	0.001 (0.015)
urban dummy		-0.029** (0.014)	-0.005 (0.014)
secondary education dummy		0.025 (0.016)	0.008 (0.017)
higher education entrance qualification dummy		0.017 (0.021)	0.007 (0.022)
university degree dummy		0.057*** (0.019)	0.038* (0.020)
self-employed dummy		0.008 (0.028)	0.055* (0.031)
manual worker dummy		-0.011 (0.018)	0.025 (0.020)
executive worker dummy		0.005 (0.021)	-0.034 (0.023)
age 65+ dummy		-0.028 (0.023)	-0.040* (0.024)
female dummy		-0.019 (0.012)	0.011 (0.012)
number of household members		0.007 (0.005)	0.011* (0.006)
income		-0.076*** (0.009)	-0.058*** (0.010)
income * income		0.004*** (0.001)	0.004*** (0.001)
Observations		3,768	3,224
*R* ^2^		0.433	0.394
Hypotheses:		Wald test p-value	
H1: no priming effect on new (marginal) AfD	A = B	0.024	0.278
H2: priming effect on new (marginal) AfD not different from the effect on strong AfD	A-B = C-D	0.115	0.074
H3: priming effect on new (marginal) AfD not different from the effect on non AfD	A-B = E-F	0.097	0.259
H4: no overall priming effect	A-B = C-D = E-F = 0	0.018	0.345

Robust errors; Coefficient estimates from a linear regression without a constant. Income is individual income measured monthly in €1,000; vaccination block is the order of additional questions on vaccination before or after the well-being questions

* *p* < 0.10

** *p* < 0.05

*** *p* < 0.01

First, we see that all AfD supporters report worse well-being than nonsupporters. We also see that, with priming, all three groups report higher deteriorations in well-being. Regarding an overall priming effect, we can reject H4. There is an overall priming effect. However, of the three groups, new AfD supporters are those whose well-being is most negatively influenced by priming, and we can reject H1. While we cannot reject the hypothesis that the effect for new AfD supporters is similar to the effect for other supporters (H2), we can reject the hypothesis that the effect is not different from the effect for non-AfD supporters (H3).

### Identity priming around the AfD federal party convention—the natural experiment

In the previous section, we presented a survey experiment that allowed us to causally evaluate an effect of populist identity priming on well-being. While such an experiment allows for clean causal attribution of the effect to the manipulation, identity priming might not work in such a structured way in the field. Also, the results that we have observed could have arisen as a statistical artefact—after all, “one swallow doesn’t make a summer” [48]. Replications of once-established findings can greatly raise confidence in their veracity [49, 50] and boost poststudy probabilities [48]. In this subsection, we ask whether we can replicate our previous results in a different—potentially more realistic—context. Such a context was delivered to us in wave 2, which allowed us to exploit the fact that the AfD federal party convention took place on November 28–29, 2020, in the middle of our sampling window, which ran from November 23 to December 14. The convention received considerable media attention, which serves as our prime.

Although the primary goal of the convention was to formulate positions regarding social policy, in particular pertaining to the pension system, the convention ended with a lively internal dispute between the moderate and the more radical wing of the party that followed after the opening address by party chairman Jörg Meuthen. This dispute was widely reported in the media. There was a spike in the number of press articles containing a phrase “AfD Parteitag” on November 29, with a similar trend in Twitter engagement [51] and in Google searches for the phrase “AfD,” which is shown in Fig A2 in the S1 File. We see that, on the first day of the AfD convention, the number of searches tripled and, on the second, it quadrupled from the average before.

Given the considerable media attention, we conjecture that the media coverage of the convention primed the identity of marginal AfD supporters. For firm AfD voters, who no longer question their support, similar priming might be weak. Given the considerable media attention, which could have affected the likelihood of the expression of support for the AfD during that time, we again defined our target group based on the previous survey wave. Because, in wave 1, we do not have a question about the length of support for the AfD, we turn to the question about the extent of support for the party manifesto. We define marginal AfD supporters as those who answered that they agree with most points (but did not choose the answer fully agree: “*stimme voll und ganz überein*”). There are 669 individuals fulfilling this criterion (and who have no missing responses for the baseline characteristics). The remaining AfD supporters are defined as other AfD supporters (324 individuals), and all others are defined as non-AfD-supporters (2,231 individuals) for the purpose of the following estimations. We created a “priming” dummy, equal to one during the two convention days and the day after (November 28–30). We also created and a “no priming” dummy, which was the reverse of the “priming” dummy. As for the measure of well-being, we again use a variable indicating average well-being (higher values correspond to lower well-being), which is calculated as the average of nonmissing values of all four main well-being questions.

We present the results in Table 1 (Column II) and visually in Fig 2 (right panel), following the approach of the controlled survey experiment. More specifically, we present coefficient estimates from an OLS regression without a constant, where each AfD status dummy (marginal, other, non AfD) is interacted with the treatment dummies for priming or no priming. We also include a baseline set of control variables. The presented coefficients show means for each status-treatment combination after correcting for any differences in observables. At the bottom of Table 1, we provide the same hypotheses tests as above (with new AfD now being replaced by marginal AfD supporters).

First, we see that AfD supporters in general report worse well-being than supporters of other parties. Second, we see that the priming effect now is no longer unidirectional. For marginal AfD supporters, we observe, on average, worse well-being during the convention than in the window outside. This difference is, however, not statistically significant (H1). On the other hand, we observe that priming through the convention had opposite effects on the remaining groups: Other AfD supporters reported a smaller deterioration in well-being during the convention while there is no discernible effect for supporters of other parties. Crucially, however, the difference between the effects on marginal and other supporters is significant (H2). In contrast, the difference between the effects on marginal supporters and nonsupporters is not statistically significant at conventional levels (H3) and there is no overall priming effect (H4). The key takeaway is that, compared to other AfD supporters, marginal supporters primed with AfD topics do experience significant relative deteriorations in well-being.

In order to make sure that the difference in the priming effect for marginal AfD supporters is not a statistical artefact, we perform the following test in the S1 File: We move our three-day-window dummy outside of the true AfD meeting (which simulates a fictitious priming window). Then, we regress the average well-being on indicator variables for the AfD meeting, marginal AfD support, and the interaction between these two variables, also including other AfD supporters and control variables. (Notice, however, that we do not estimate the priming effect on other groups in this exercise.) For the actual days of the meeting, we report the results in Table A2 in S1 File. There, we see that marginal AfD supporters report lower average well-being around the AfD convention, and this coefficient is significant at p < 0.05. As shown in Fig A2 in S1 File, we see that the coefficients obtained when the window is close to the true timing of the meeting are higher, while the coefficients obtained when the window is further away from the AfD meeting are lower and turn insignificant. We also repeat the exercise for each separate well-being variable and find a similar, if slightly weaker, effect (not presented).

Overall, we conclude that the party convention caused marginal AfD supporters to report significantly worse well-being in comparison to the changes observed for other AfD supporters. The fact that we observe different results for the other groups than in the controlled survey experiment is not surprising as we do not have perfect control in the natural setting and supporters of other parties do not receive a prime tailored to them.

### Supplementary evidence: Correlation between well-being and AfD support across all four survey waves

Our experiments show a causal link between AfD support and diminished well-being, in particular for new and marginal supporters. If this is not just an artefact of the specific primings, we would expect to find a relationship between AfD support and well-being across all our survey waves. In what follows, we will document such correlational evidence of changes in AfD support with changes in well-being. In fact, the correlation is strong and survives when we control for socio-economic and other individual characteristics.

In Table 2, we regress our well-being variable on dummy variables that indicate four types of support for the AfD. Those types now include: new AfD voters (individuals in the current wave who indicate intending to vote for the AfD while in the previous wave they intended to vote for another party), past AfD supporters (the reverse of the former), steady AfD supporters (who indicated intending to vote for the AfD in the current and in the previous wave), with steady nonsupporters (who did not intend to vote for the AfD in either wave) as the omitted baseline. There are 75 new AfD supporters in Wave 2, 40 in wave 3 and 62 in wave 4. The respective numbers for past AfD supporters are 223, 97, and 152 and for steady AfD supporters 661, 461, and 1,089. Thus, we regress a subjectively reported change in well-being (current or expected in the future) on a change in AfD support.

**Table 2:** AfD status and well-being.

Outcome variable:	Feeling worse score (average over four well-being questions)		
	(I) OLS regression	(II) OLS with control variables	(III) including time and individual fixed effects
New AfD	0.159*** (0.029)	0.155*** (0.032)	0.091*** (0.031)
Past AfD	0.077*** (0.017)	0.082*** (0.018)	0.023 (0.019)
Steady AfD	0.166*** (0.009)	0.160*** (0.010)	0.017 (0.028)
Wave 3	-0.040*** (0.009)	-0.031*** (0.009)	-0.042*** (0.007)
Wave 4	0.035*** (0.009)	0.039*** (0.009)	0.011 (0.007)
age		0.011*** (0.002)	
age * age		-0.000*** (0.000)	
eastern German states dummy		-0.021** (0.010)	
Berlin dummy		0.003 (0.019)	
suburban dummy		-0.003 (0.010)	
urban		-0.027*** (0.010)	
secondary education dummy		0.014 (0.012)	
higher education entrance qualification dummy		0.007 (0.015)	
university degree dummy		0.036*** (0.013)	
self-employed dummy		0.012 (0.019)	
manual worker dummy		-0.019 (0.013)	
executive worker dummy		-0.005 (0.015)	
age 65+ dummy		-0.029* (0.015)	
female dummy		-0.012 (0.008)	
number of household members		0.007* (0.004)	
income		-0.071*** (0.006)	
income * income		0.005*** (0.001)	
Constant	0.191*** (0.006)	0.002 (0.052)	0.227*** (0.009)
Observations	8149	7219	6012
*R* ^2^	0.060	0.090	0.749

Well-being measured in Waves 2–4; Robust errors

* *p* < 0.10

** *p* < 0.05

*** *p* < 0.01

The estimates stem from a simple linear probability model, meaning that coefficients are easy to interpret. We present the results in three columns. (I) without control variables, (II) with baseline control variables, and (III) accounting for individual fixed effects.

In column (I) the constant shows the average well-being among non AfD supporters. All three AfD coefficients are positive and statistically significant, indicating that all these groups report deteriorations in well-being more often than supporters of other parties. While the coefficients on new and steady AfD support are similar in magnitude, the coefficient on past AfD support is less than half of their size.

Column (II) includes a number of individual characteristics. While the above-reported coefficients change only slightly and remain highly significant, we can report on a number of interesting correlations. Older individuals are gloomier, but this correlation slows down over time (the quadratic term is significant and negative and the dummy on individuals aged 65+ is significant and negative). Also, individuals living in cities and in the eastern German states indicate higher well-being, and those with the highest education level indicate diminished personal well-being more often. Individuals living in larger households report lower well-being. However, the only other variable with significance levels and magnitudes similar to the AfD status variable is personal income. As expected, more income goes hand in hand with improved well-being, but money is shown to have diminishing returns. Back-of-the-envelope calculations suggest that it would take an additional monthly income of more than €2,500 to offset the difference between AfD supporters and supporters of other parties. Regarding changes over time, we observe again that personal well-being improves before the elections in 2021, while future expectations decrease in December 2021. Finally, in Column (III) with individual fixed effects, only the coefficient on new AfD remains statistically significant, indicating a robust relationship between turning to the AfD and reporting diminished well-being. We conclude that the correlation between feelings of misery and AfD support is hence not only a kind of snapshot—it holds dynamically. Switching to the AfD goes hand in hand with an increase in feelings of misery and switching away is associated with an improvement in outlook.

In the S1 File, we provide additional analysis (i) separately for all variables entering our well-being score and, in addition, (ii) for two questions about long-term financial security that we have included in wave 1 and wave 4 of our survey. The conclusions remain similar.

## Discussion

A substantial body of literature, both theoretical and empirical, addresses the causes of political support for right-wing populist movements. The leading strands of theory focus on broad cultural drivers, including xenophobia [52, 53] and on economic causes, in particular (relative) income losses due to globalization [39]. While both theories have found empirical support in some countries, Germany is a notable exception with respect to the “losers of globalization” theory: for the German AfD, there is broad agreement in the literature that neither variables related to low socio-economic status, such as education, income, occupational prestige, or unemployment, nor subjective perceptions of one’s adverse personal economic situation are related to AfD support [40–45]. Rather, societal discontent, dissatisfaction with democracy, and anti-immigration attitudes emerge as key drivers of right-wing voting in Germany [44, 45, 54–60].

In this study, we depart from the previous literature, which invariably depicts economic circumstances and perceptions as possible drivers of political preferences, and theorize that the arrow of causality could actually go in the opposite direction. Our findings challenge the prevailing assumption that causality moves unidirectionally from life dissatisfaction to support for populist parties.

We hypothesized that the adverse effects of taking a stance for the AfD is driven by the rhetoric of negativity the party employs. But there might also be other potential explanations for the observed pattern. As shown in a cross-country study by Owen, Videras, and Willemsen [61], supporters of minority political parties report lower life satisfaction when their country has a parliamentary system; in other words, when minority status is salient. Similarly, Di Tella and MacCulloch [62] show that individuals report higher levels of well-being when the party they support is in power, even after controlling for political outcomes. It is hence also plausible that minority status explains our result: that is, that German voters who self-select into a minority party such as the AfD experience diminished well-being. However, this theory does not explain why the effect would be strongest for new and marginal AfD supporters.

## Conclusions

In this study, we establish a causal link revealing that individuals who are new or marginal supporters of the AfD exhibit deterioration in well-being. We establish this through two priming experiments, in which new and marginal supporters react differently to the prime than other supporters. In addition, we establish a strong correlation between negative perceptions of personal and financial well-being and support for the German right-wing populist party AfD. These are novel findings, since previous studies (i) typically conjectured the arrow of causality going in the opposite direction; and (ii) rarely found any association between well-being and AfD support.

We believe that our correlational evidence differs from that of most previous research because of our choice of a measurement tool for well-being that draws on intra- rather than interpersonal comparisons. It avoids comparisons with others and establishes a clear reference point. As AfD supporters are no poorer than the supporters of other parties, interpersonal comparisons may miss changes in private perceptions, and without clearly specified reference points, interpersonal measurements may suffer from substantial noise. Unlike the previous literature, we uncover a stable and economically meaningful association between AfD support and feelings of deprivation.

The biggest caveat when interpreting our results is that we cannot exclude a causal link from discontent to AfD support. As the literature has shown [44, 45, 54–60], discontent with the political system is an important driver of support for right-wing populist movements, in Germany and elsewhere. So, voters who experience discontent may select themselves into the group of AfD supporters. But that does not improve their well-being. In contrast, personal discontent increases through selection into the exposure to a rhetoric of negativity (or into a minority). This subtlety should be kept in mind in discussions of the relationship between well-being and AfD support.

Our findings are new, and we believe they point to an important implication for the resilience of democracy. When supporters of extremist movements perceive a decline in well-being due to the negative rhetoric of their chosen party, it may be easier to bring them back into the mainstream by focusing on different topics, not reiterating the anxiety loaded themes pushed by right wingers. Similarly, acting swiftly might be a good idea, as we see the negative effects on well-being diminish when voters establish a fully-fledged identity as right-wing supporters.

## Supporting information

S1 File Appendix. (DOCX)
