# Supplementary material for: Support for a right-wing populist party and subjective well-being: Experimental and survey evidence from Germany
Source: PLoS One. 2024 Jun 26;19(6):e0303133. doi: 10.1371/journal.pone.0303133 (PMC11207160; doi:10.1371/journal.pone.0303133)
Supplement: S1 File — (DOCX) [file pone.0303133.s001.docx]

# **Appendix for**

# **Support for the German right-wing populist party AfD diminishes subjective well-being – Survey and experimental evidence**

# **Appendix section A: Additional tables and figures**

**Table A1. Descriptive statistics of baseline variables by wave.**

|  | wave 1: Nov/Dec 2019 | | wave 2: Nov/Dec 2020 | | wave 3: Aug/Sep 2021 | | wave 4: Nov/Dec 2021 | |
| --- | --- | --- | --- | --- | --- | --- | --- | --- |
|  | mean | std. err. | mean | std. err. | mean | std. err. | mean | std. err. |
| AfD vote intention^a^ | 0,249 | 0,006 | 0,204 | 0,006 | 0,314 | 0,007 | 0,304 | 0,007 |
| own economic situation in the 90's better than now | 0,330 | 0,007 |  |  |  |  | 0,304 | 0,007 |
| parents' economic situation (at the same age) better that own now | 0,316 | 0,007 |  |  |  |  | 0,527 | 0,009 |
| personally worse off than last year |  |  | 0,314 | 0,008 | 0,229 | 0,006 | 0,368 | 0,008 |
| financially worse off than last year |  |  | 0,251 | 0,007 | 0,254 | 0,006 | 0,305 | 0,007 |
| expect to be worse off personally in the coming year |  |  | 0,165 | 0,006 | 0,172 | 0,006 | 0,286 | 0,007 |
| expect to be worse off financially in the coming year |  |  | 0,225 | 0,007 | 0,251 | 0,006 | 0,240 | 0,007 |
| feeling worse score (average over four well-being questions) |  |  | 0,227 | 0,005 | 0,217 | 0,004 | 0,276 | 0,005 |
| age | 51,388 | 0,222 | 54,657 | 0,238 | 55,667 | 0,201 | 56,314 | 0,211 |
| age 65+ dummy | 0,246 | 0,006 | 0,296 | 0,007 | 0,299 | 0,006 | 0,310 | 0,007 |
| female dummy | 0,501 | 0,007 | 0,492 | 0,008 | 0,441 | 0,007 | 0,440 | 0,008 |
| lower secondary education dummy | 0,162 | 0,005 | 0,165 | 0,006 | 0,156 | 0,005 | 0,162 | 0,006 |
| secondary education dummy | 0,475 | 0,007 | 0,481 | 0,008 | 0,455 | 0,007 | 0,452 | 0,008 |
| higher education entrance qualification dummy | 0,129 | 0,005 | 0,122 | 0,005 | 0,126 | 0,005 | 0,119 | 0,005 |
| university degree dummy | 0,215 | 0,006 | 0,222 | 0,007 | 0,255 | 0,006 | 0,261 | 0,007 |
| single dummy | 0,274 | 0,006 | 0,282 | 0,007 | 0,288 | 0,006 | 0,290 | 0,007 |
| not in workforce dummy | 0,466 | 0,007 | 0,480 | 0,008 | 0,474 | 0,007 | 0,485 | 0,008 |
| self-employed dummy | 0,051 | 0,003 | 0,050 | 0,003 | 0,054 | 0,003 | 0,050 | 0,003 |
| manual worker dummy | 0,114 | 0,004 | 0,113 | 0,005 | 0,127 | 0,005 | 0,127 | 0,005 |
| executive worker dummy | 0,070 | 0,004 | 0,069 | 0,004 | 0,078 | 0,004 | 0,080 | 0,004 |
| employed in for-profit sector dummy | 0,341 | 0,007 | 0,337 | 0,008 | 0,344 | 0,008 | 0,343 | 0,009 |
| employed in public service dummy | 0,101 | 0,004 | 0,103 | 0,005 | 0,101 | 0,005 | 0,100 | 0,005 |
| employed in non-profit sector dummy | 0,021 | 0,002 | 0,022 | 0,002 | 0,024 | 0,003 | 0,023 | 0,003 |
| urban dummy | 0,390 | 0,007 | 0,381 | 0,008 | 0,373 | 0,007 | 0,377 | 0,007 |
| suburban dummy | 0,305 | 0,006 | 0,304 | 0,007 | 0,311 | 0,007 | 0,310 | 0,007 |
| rural dummy | 0,299 | 0,006 | 0,312 | 0,007 | 0,316 | 0,007 | 0,312 | 0,007 |
| eastern German states dummy | 0,185 | 0,005 | 0,190 | 0,006 | 0,217 | 0,006 | 0,210 | 0,006 |
| Berlin dummy | 0,049 | 0,003 | 0,049 | 0,003 | 0,053 | 0,003 | 0,053 | 0,003 |
| number of household members | 2,240 | 0,017 | 2,169 | 0,019 | 2,137 | 0,015 | 2,116 | 0,016 |
| N | 5,078 |  | 3,864 |  | 5,061 |  | 4,342 |  |
| Retention rate relative to last wave |  |  | 0.761 |  | 0.869 |  | 0.858 |  |
| New sampling (N) |  |  | - |  | 1,702 |  |  | - |

The variable AfD vote intention was collected in wave 1 and 2 with the following questions: “If elections were next Sunday.” In wave 3, we asked for the vote intention in the upcoming 2021 parliamentary elections and in wave 4 we asked how the individuals votes in those elections. Baseline characteristics were only collected in wave 1, 3, and 4.

**Table A2. The effect of the AfD meeting.**

Dependent variable: low well-being score

| marginal AfD supporter | 0.007 (0.018) |
| --- | --- |
| AfD meeting | -0.020 (0.013) |
| marginal AfD supporter x AfD meeting | 0.064^**^ (0.033) |
| steady AfD supporter | 0.125^***^ (0.017) |
| Observations | 3,802 |
| *R*^2^ | 0.033 |

Controls: steady AfD dummy, eastern German states dummy, female dummy, age, age 65+ dummy, suburban dummy, urban dummy, more than 3 household members dummy, constant; ^*^ *p* < 0.10, ^**^ *p* < 0.05, ^***^ *p* < 0.01

**Table A3. Support for AfD policies.**

|  | **N** | **Mean** | **Std. dev.** | **Min.** | **Max.** |
| --- | --- | --- | --- | --- | --- |
| support for AfD EU policy | 1,914 | 0.763 | 0.424 | 0 | 1 |
| support for AfD migration policy | 1,953 | 0.967 | 0.179 | 0 | 1 |
| support for AfD COVID-19 policy | 1,949 | 0.603 | 0.489 | 0 | 1 |
| support for AfD democracy policy | 1,918 | 0.961 | 0.193 | 0 | 1 |
| support for AfD finance policy | 1,917 | 0.763 | 0.426 | 0 | 1 |
| support for AfD climate policy | 1,881 | 0.785 | 0.411 | 0 | 1 |
| support for AfD transportation policy | 1,939 | 0.790 | 0.408 | 0 | 1 |

**Fig A1. Google trends search frequency of “AfD” over time of the survey**.


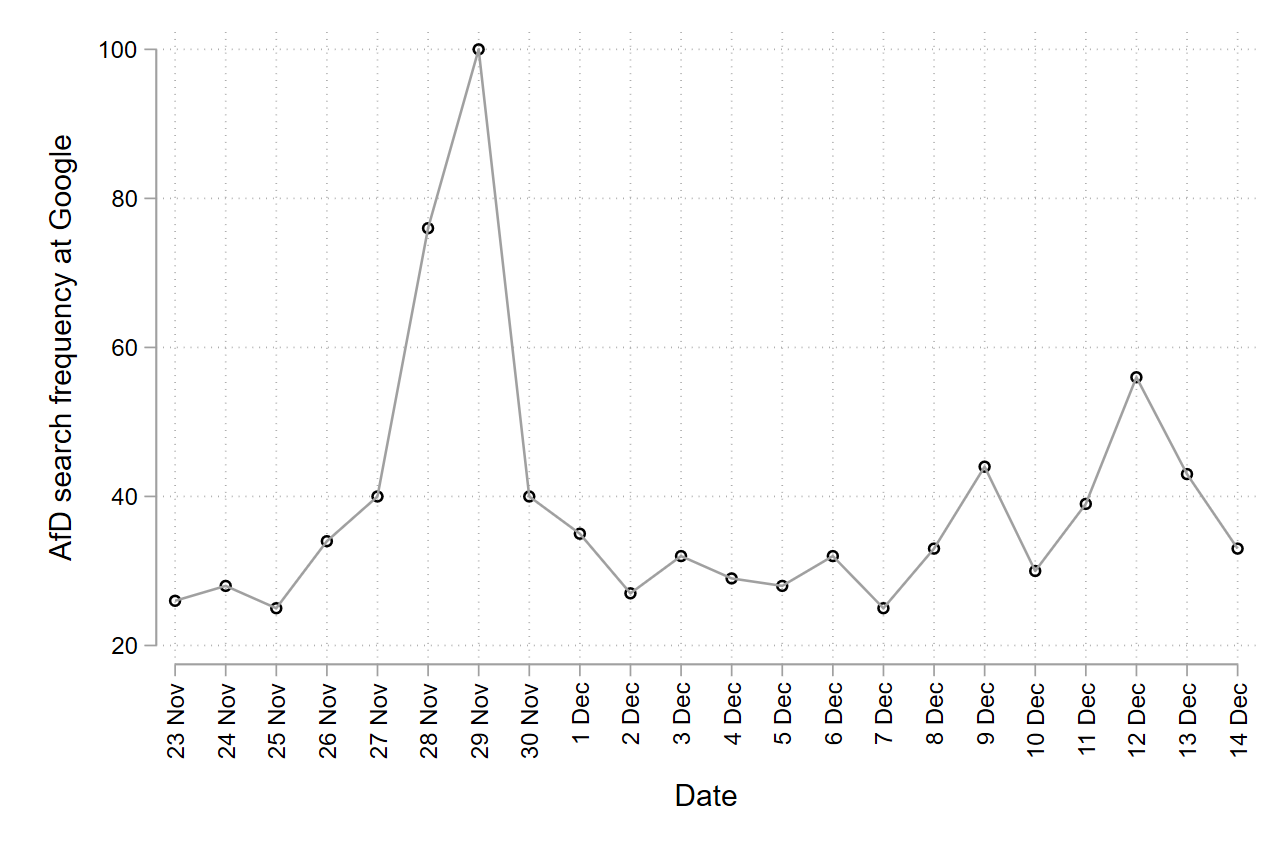


Search frequency at Google for the phrase “AfD.”

**Fig A2. Placebo exercise: the effect of a fictious and true AfD party meeting on average well-being indicated by marginal AfD supporters.**

*
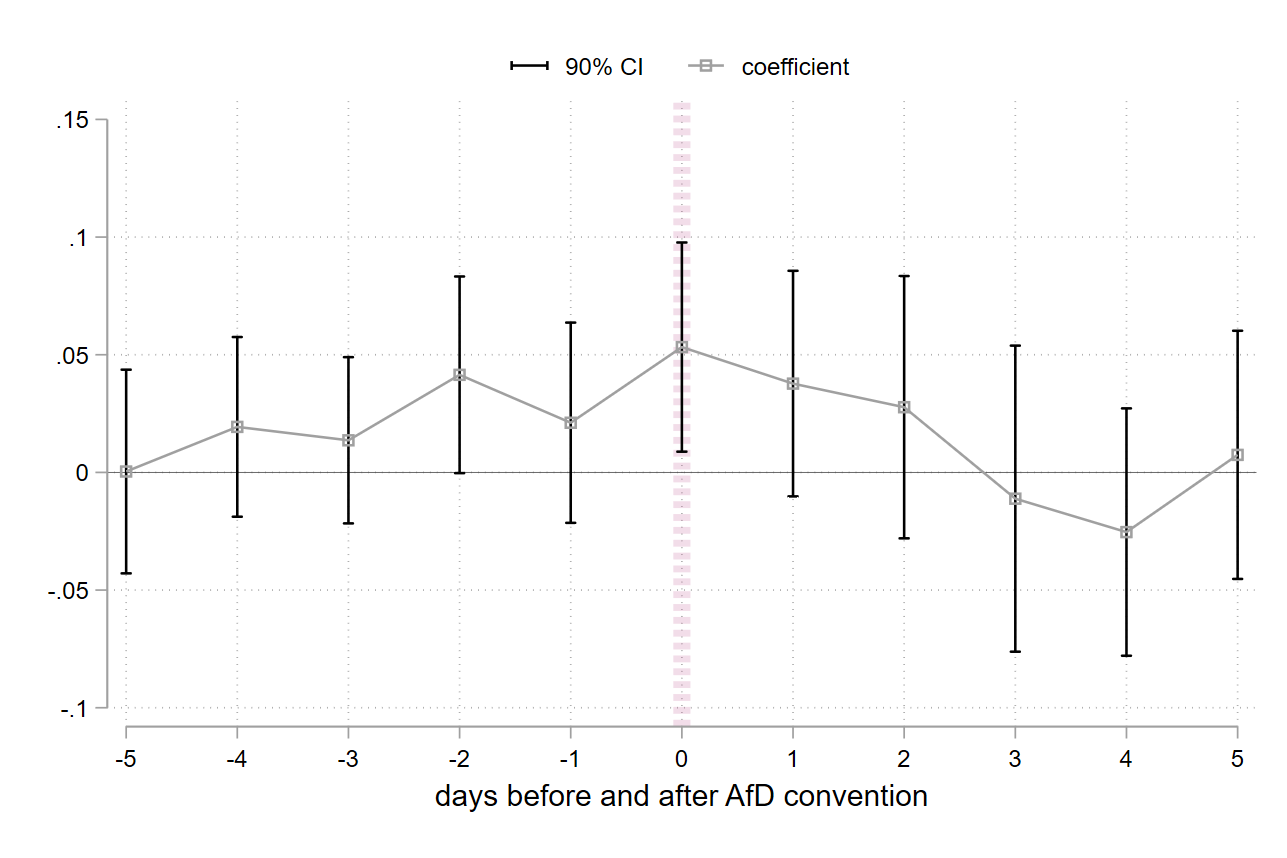
*

We use a 3-day window. The horizontal axis denotes the starting day of the window relative to the first AfD meeting day.

**Table A4. AfD status and well-being: Compared with last year and expectations for the future.**

| Outcome variable: | | **(I) Personally worse off than last year** | | **(II) Financially worse off than last year** | | **(III) Expect to be worse off personally in the coming year** | | **(IV) Expect to be worse off financially in the coming year** | |
| --- | --- | --- | --- | --- | --- | --- | --- | --- | --- |
|  | | Panel A: OLS regression | | | | | | | |
| New AfD | | 0.169^***^ (0.037) | | 0.172^***^ (0.037) | | 0.147^***^ (0.035) | | 0.141^***^ (0.036) | |
| Past AfD | | 0.079^***^ (0.022) | | 0.063^***^ (0.021) | | 0.082^***^ (0.020) | | 0.092^***^ (0.022) | |
| Steady AfD | | 0.133^***^ (0.012) | | 0.133^***^ (0.012) | | 0.195^***^ (0.011) | | 0.212^***^ (0.012) | |
| Wave 3 | | -0.109^***^ (0.012) | | -0.015 (0.012) | | -0.017 (0.010) | | -0.006 (0.012) | |
| Wave 4 | | -0.008 (0.012) | | 0.026^**^ (0.011) | | 0.060^***^ (0.010) | | 0.074^***^ (0.011) | |
| Constant | | 0.262^***^ (0.009) | | 0.205^***^ (0.009) | | 0.107^***^ (0.007) | | 0.166^***^ (0.008) | |
| Observations | | 8,117 | | 8,110 | | 7,643 | | 7,808 | |
| *R*^2^ | | 0.032 | | 0.022 | | 0.061 | | 0.057 | |
|  | | Panel B: OLS with control variables | | | | | | | |
| New AfD | 0.169^***^ (0.039) | | 0.159^***^ (0.039) | | 0.148^***^ (0.037) | | 0.138^***^ (0.039) | |  |
| Past AfD | 0.085^***^ (0.024) | | 0.074^***^ (0.022) | | 0.080^***^ (0.021) | | 0.087^***^ (0.023) | |  |
| Steady AfD | 0.132^***^ (0.013) | | 0.130^***^ (0.013) | | 0.181^***^ (0.012) | | 0.203^***^ (0.013) | |  |
| Wave 3 | -0.098^***^ (0.013) | | 0.001 (0.013) | | -0.014 (0.011) | | -0.002 (0.012) | |  |
| Wave 4 | -0.003 (0.013) | | 0.035^***^ (0.012) | | 0.059^***^ (0.011) | | 0.076^***^ (0.012) | |  |
| age | 0.006^**^ (0.003) | | 0.013^***^ (0.003) | | 0.010^***^ (0.002) | | 0.015^***^ (0.003) | |  |
| age * age | -0.000^**^ (0.000) | | -0.000^***^ (0.000) | | -0.000^***^ (0.000) | | -0.000^***^ (0.000) | |  |
| eastern German states dummy | -0.057^***^ (0.013) | | -0.035^***^ (0.013) | | 0.001 (0.013) | | 0.012 (0.014) | |  |
| Berlin dummy | 0.006 (0.025) | | 0.003 (0.024) | | -0.005 (0.022) | | 0.011 (0.024) | |  |
| suburban dummy | 0.000 (0.013) | | 0.000 (0.013) | | -0.014 (0.012) | | 0.001 (0.013) | |  |
| urban | -0.024^*^ (0.013) | | -0.024^*^ (0.012) | | -0.025^**^ (0.011) | | -0.033^***^ (0.013) | |  |
| secondary education dummy | 0.040^***^ (0.015) | | 0.009 (0.015) | | 0.005 (0.014) | | 0.007 (0.015) | |  |
| higher education entrance qualification dummy | 0.024 (0.020) | | -0.001 (0.020) | | -0.004 (0.018) | | 0.017 (0.020) | |  |
| university degree dummy | 0.065^***^ (0.018) | | 0.029 (0.017) | | 0.025 (0.016) | | 0.024 (0.017) | |  |
| self-employed dummy | 0.003 (0.026) | | 0.082^***^ (0.026) | | -0.016 (0.022) | | -0.020 (0.025) | |  |
| manual worker dummy | -0.023 (0.017) | | -0.014 (0.017) | | -0.016 (0.015) | | -0.015 (0.017) | |  |
| executive worker dummy | -0.004 (0.020) | | -0.005 (0.019) | | 0.018 (0.018) | | -0.012 (0.019) | |  |
| age 65+ dummy | -0.022 (0.021) | | -0.016 (0.020) | | -0.036^*^ (0.019) | | -0.041^**^ (0.021) | |  |
| female dummy | -0.001 (0.011) | | -0.019^*^ (0.011) | | -0.009 (0.010) | | -0.022^**^ (0.011) | |  |
| number of household members | 0.002 (0.005) | | 0.009^*^ (0.005) | | 0.008^*^ (0.004) | | 0.008^*^ (0.005) | |  |
| income | -0.066^***^ (0.009) | | -0.088^***^ (0.008) | | -0.053^***^ (0.008) | | -0.076^***^ (0.008) | |  |
| income * income | 0.005^***^ (0.001) | | 0.005^***^ (0.001) | | 0.004^***^ (0.001) | | 0.005^***^ (0.001) | |  |
| Constant | 0.216^***^ (0.078) | | 0.015 (0.074) | | -0.122^**^ (0.060) | | -0.172^**^ (0.069) | |  |
| Observations | 7,197 | | 7,196 | | 6,821 | | 6,962 | |  |
| *R*^2^ | 0.047 | | 0.057 | | 0.073 | | 0.086 | |  |
|  | | Panel C: including time and individual fixed effects | | | | | | | |
| New AfD | | 0.165^***^ (0.051) | | 0.049 (0.043) | | 0.115^**^ (0.045) | | 0.055 (0.045) | |
| Past AfD | | 0.031 (0.031) | | -0.006 (0.028) | | 0.042 (0.026) | | 0.029 (0.028) | |
| Steady AfD | | 0.070 (0.044) | | -0.057 (0.036) | | 0.035 (0.038) | | 0.009 (0.040) | |
| Wave 3 | | -0.101^***^ (0.011) | | -0.019^**^ (0.010) | | -0.018^*^ (0.009) | | -0.008 (0.010) | |
| Wave 4 | | -0.024^**^ (0.012) | | 0.004 (0.010) | | 0.029^***^ (0.010) | | 0.043^***^ (0.011) | |
| Constant | | 0.270^***^ (0.015) | | 0.254^***^ (0.013) | | 0.145^***^ (0.013) | | 0.219^***^ (0.014) | |
| Observations | | 5,989 | | 5,989 | | 5,568 | | 5,727 | |
| *R*^2^ | | 0.603 | | 0.685 | | 0.644 | | 0.689 | |

Well-being measured in Waves 2–4; Robust errors; ^*^ *p* < 0.10, ^**^ *p* < 0.05, ^***^ *p* < 0.01

In Column I and II, we look at retrospective evaluations of well-being. In Panel A, the constant shows the shares of non-AfD voters reporting diminished well-being in 2020, they are as high as 21–26%, presumably due to the pandemic. However, steady AfD supporters are more likely to report reductions in personal and financial well-being. The size of the coefficient suggests an additional 13 percentage points, translating into a relative difference of around 50%. These differences are significant at the 1% level. For new AfD supporters the shares reporting reduction in well-being are even larger, an additional 16–17 percentage points, translating into a relative difference of 60–70%. This difference, too, is significant at the 1% level. Former AfD supporters also differ from non-AFD supporters, but the differences are half as large as those for steady AfD supporters.

In Column III and IV, we look at the expectations for the future development of well-being. We find a similar pattern. Among non-AfD supporters, 11–17% have low expectations for the future and this drift is enhanced for AfD supporters. Steady AfD supporters, are on average, 20 to 21 percentage points more likely to expect a downward trend than non-AfD supporters, which equates to relative difference of more than 100%. These differences are again significant at the 1% level. For new AfD supporters the likelihood of low expectations is, again, higher by 14 to 15 percentage points, also significant at the 1% level. Former AfD supporters, on the other hand, exhibit a smaller tendency towards negative expectations but higher than the non-AfD supporters, with coefficients of around 8% to 9%, also significant at the 1% level.

Over time, we see an increase in personal well-being in Wave 3 (before the elections in 2021), while future expectations drop on average in Wave 4 .

Panel B includes a number of individual characteristics. While the above-reported coefficients change only slightly and remain highly significant, we can report on a number of interesting correlations. Older individuals are gloomier, but this correlation slows down over time (quadratic term is significant as well and the dummy on individuals aged 65+ is significant in case of future expectations). Also, individuals living in cities indicate higher well-being, and those with highest education level indicate diminished personal well-being more often. However, the only other variable with consistently significant levels similar to the AfD status variable is personal income. As expected, more income goes hand in hand with improved well-being, but money is shown to have diminishing returns. Back-of-the-envelope calculations suggest that it would take an additional monthly income of more than €3,000 to offset the difference between the supporters of AfD and the supporters of other parties. Regarding changes over time, we observe again that personal well-being gets better before the elections in 2021, while future expectations decrease in December 2021.

Panel C takes the analysis a step further by accounting for fixed individual characteristics, considering, for example, overall higher pessimism. Note that, in this case, we can still estimate the coefficient for steady AfD support, because we define steady AfD support based on the current and the previous wave and not across all waves. The presented constant is the average of all fixed effects. We now find being a new AfD supporters increases the probability for reporting diminished personal well-being by 17 percentage points, significant at the 1% level. For expectations about the future the difference is 12 percentage points, also significant at 5% level. All other coefficients are not significant.

**Table A5. AfD status and well-being: Comparisons with parents and the 90’s.**

|  | **Parents' economic situation (at the same age) better that own now** | | | **Own economic situation in the 90's better than now** | | |
| --- | --- | --- | --- | --- | --- | --- |
| New AfD | 0.102^***^ (0.035) | 0.100^***^ (0.035) | 0.094 (0.067) | 0.120^***^ (0.040) | 0.107^***^ (0.041) | 0.065 (0.066) |
| Former AfD | 0.071^**^ (0.031) | 0.091^***^ (0.032) | 0.089^*^ (0.051) | 0.068^*^ (0.036) | 0.050 (0.038) | -0.028 (0.069) |
| Steady AfD | 0.101^***^ (0.013) | 0.097^***^ (0.014) | 0.080^*^ (0.043) | 0.149^***^ (0.015) | 0.136^***^ (0.016) | 0.064 (0.050) |
| Wave 4 | 0.039^***^ (0.012) | 0.041^***^ (0.012) | 0.016 (0.012) | 0.023^*^ (0.014) | 0.024 (0.014) | -0.009 (0.015) |
| age |  | 0.023^***^ (0.003) |  |  | 0.040^***^ (0.010) |  |
| age * age |  | -0.000^***^ (0.000) |  |  | -0.000^***^ (0.000) |  |
| eastern German states dummy |  | -0.110^***^ (0.015) |  |  | -0.055^***^ (0.019) |  |
| Berlin dummy |  | 0.031 (0.030) |  |  | 0.021 (0.032) |  |
| suburban dummy |  | 0.007 (0.015) |  |  | 0.018 (0.018) |  |
| urban dummy |  | 0.002 (0.015) |  |  | 0.005 (0.018) |  |
| secondary education dummy |  | 0.030^*^ (0.018) |  |  | 0.004 (0.021) |  |
| higher education entrance qualification dummy |  | 0.048^**^ (0.024) |  |  | 0.011 (0.029) |  |
| university degree dummy |  | 0.066^***^ (0.020) |  |  | -0.043^*^ (0.024) |  |
| self-employed dummy |  | 0.089^***^ (0.030) |  |  | 0.048 (0.033) |  |
| manual worker dummy |  | -0.032 (0.020) |  |  | -0.030 (0.025) |  |
| executive worker dummy |  | -0.013 (0.023) |  |  | -0.028 (0.031) |  |
| age 65+ dummy |  | -0.049^**^ (0.024) |  |  | -0.040 (0.027) |  |
| female dummy |  | -0.019 (0.013) |  |  | -0.023 (0.016) |  |
| number household members |  | -0.020^***^ (0.006) |  |  | -0.019^**^ (0.008) |  |
| income |  | -0.116^***^ (0.010) |  |  | -0.119^***^ (0.012) |  |
| income * income |  | 0.008^***^ (0.001) |  |  | 0.007^***^ (0.001) |  |
| Constant | 0.278^***^ (0.009) | -0.032 (0.079) | 0.284^***^ (0.017) | 0.439^***^ (0.011) | -0.598^*^ (0.305) | 0.475^***^ (0.019) |
| Observations | 6,480 | 5,801 | 3,464 | 5,059 | 4,510 | 2,954 |
| *R*^2^ | 0.012 | 0.073 | 0.723 | 0.020 | 0.078 | 0.712 |

Well-being measured in wave 1 and 4; The question on the comparisons with the 90s has only been asked to individuals born before 1976. Robust errors; ^*^ *p* < 0.10, ^**^ *p* < 0.05, ^***^ *p* < 0.01

In order to delve deeper into the connection between AfD support and well-being, we now turn to data from wave 1 of our survey from December 2019 and wave 4 in December 2021, which also included two questions about long-term financial security. In waves 1 and 4, we asked participants how they compare their economic well-being to that of their parents at their age (“When your parents were as old as you are today, how did your parents fare economically compared to you today? Please think of the overall economic situation, including all types of income and assets.”). Another question referred to economic well-being in comparison to the 1990s (“If you think back: how did you fare economically in the 90s compared to today? Please consider your entire economic situation, including all types of income and assets.”). For both questions we created a dummy variable equal to one in case the respondents chose ‘better’ and ‘much better’ meaning that they judge their present situation to be worse than in the past.

We essentially repeat the above analysis with the long-term measures: comparisons to one’s own parents’ well-being and comparisons to the 1990s. Regarding changes in voting intentions, for Wave 1, we rely on stated voting in the elections of 2017, as there is no previous wave on which we could draw. For Wave 4, we rely on voting intentions before the elections in 2021 (that is, the data from Wave 3) as we did for the previous analysis. Table 2 shows the results for comparisons with parents (Columns I–III) and with the 1990s (Columns IV–VI) using simple OLS regressions without controls (Column I and IV) or with controls (Column II and V), and including individual fixed effects (Column III and VI). In cross-sectional regressions, we found very similar results to Table A4. Compared to steady non-supporters, new and steady AfD supporters express the highest deterioration in their well-being, followed by past supporters. Different feelings of relative deprivation over decades may, of course, again stem from differences in socio-economic characteristics, which is why we control for these in Columns II and V. But, as in our first analysis, the estimated coefficients for the AfD dummies remain virtually identical. While there is a whole array of socio-economic factors that help explain feelings of relative deprivation, there is a correlation with AfD support over and above these factors. Once again, we compared the size of these associations to the associations between the well-being and income. We find that (steady) AfD voters would have to earn around €1,000 per month in additional income to ensure similar levels of well-being as of non-AfD supporters, both with respect to the comparisons with the 1990s and their parents.

When accounting for individual fixed effects in Column III, the coefficients remain similar but mostly loose significance. In Column VI all coefficients turn insignificant. Note that the sample here is somewhat smaller and we are looking at a kind of a double difference what might be anyhow difficult to interpret.

# **Appendix section B: Questionnaire**

Questions in the experiment in Wave 4 pertaining to the section on priming and the AfD (translation):

**Table B1. Support for AfD policies.**

| Questions | Which specific AfD program points for the 2021 federal election do you support?   - Germany should leave the European Union. (EU] - There are to be border controls again as well as physical barriers at the borders. At the same time, there is to be a "deportation offensive." (Migration Policy) - All measures to fight pandemics are to be abolished with immediate effect and it is to be left up to the individual citizens* to what extent they protect themselves. (Corona policy) - There should be referendums according to the Swiss model in Germany. (Democracy) - Germany should leave the Euro. (Finance) - CO2 emissions shall not be brought to zero and the conversion of industry and society to this goal shall be stopped. (Climate policy) - There should be no speed limit, no restrictions on diesel vehicles and environmental zones should be abolished. (Transport policy) |
| --- | --- |
| Answers | <1> I do not support this program item at all.  <2> I rather do not support this program point  <3> I support this program point partially  <4> I fully support this program item  <977> Don't know/ don't specify |

**Table B2. Reasons for AfD support.**

| Questions | Are there any other reasons why you support the AfD or you can imagine supporting the AfD? |
| --- | --- |
| Answers | [open text field] |

**Table B3. Support for AfD politicians.**

| Questions | How good do you find the work of the following politicians of the AfD?   - Jörg Meuthen - Alice Weidel - Björn Höcke |
| --- | --- |
| Answers | <1> Very good  <2> Good  <3> Partly partly  <4> Bad  <5> Very bad  <977> Don't know/ don't specify |

Table 4: Influence of AfD politicians.

| Questions | Should these politicians have more or less influence in the party?   - Jörg Meuthen - Alice Weidel - Björn Höcke |
| --- | --- |
| Answers | <1> More influence  <2> Just as much influence  <3> Less influence  <977> Don't know/ no answer |
